# The association of visceral adiposity index and diabetic kidney disease in elderly patients with type 2 diabetes mellitus: a cross-sectional study

**DOI:** 10.3389/fnut.2025.1556886

**Published:** 2025-06-20

**Authors:** Mengdie Chen, Yiyun Wang, Ping Feng, Lijing Wu, Chaoyin Lu, Yao Liang, Mengyao Yang, Qidong Zheng

**Affiliations:** ^1^Department of Endocrinology, Taizhou Central Hospital (Taizhou University Hospital), Taizhou, China; ^2^Department of Internal Medicine, Yuhuan Second People’s Hospital, Yuhuan, China

**Keywords:** visceral adiposity index, type 2 diabetes, diabetic kidney disease, elderly, obesity

## Abstract

**Background:**

Research on the relationship between visceral adiposity index (VAI) and diabetic kidney disease (DKD) in elderly populations remains limited. This study aimed to investigate the potential link between the VAI and DKD in elderly patients with type 2 diabetes mellitus (T2DM).

**Methods:**

Overall, this cross-sectional analysis included 2,695 older individuals with T2DM from the National Metabolic Management Center (MMC) at Yuhuan Second People’s Hospital and Taizhou Central Hospital (Taizhou University Hospital) from September 2017 to May 2024. VAI was utilized as both a continuous and categorical variable with division into tertiles. Logistic regression and smooth curve fitting were employed, together with further stratified and interaction analyses.

**Results:**

This relationship was consistently observed across analyzed subgroups. The prevalence of DKD was significantly elevated in top tertile of VAI (T3) relative to the lowest (T1) (57.6% vs. 48.1%, *p* < 0.001). Following confounder adjustment, elevated VAI was linked with a higher risk of developing DKD. Each 1-unit rise in VAI (as a continuous variable) was related to a 4% greater risk of DKD (OR = 1.04, 95% CI: 1.01–1.08, *p* = 0.015). When VAI was categorized into tertiles, individuals in T3 showed a 1.29-fold greater risk of DKD compared with cases in T1 (OR = 1.29, 95% CI: 1.05–1.59, *p* = 0.015). A marked positive link was seen between VAI and DKD risk in all three regression models (P for trend < 0.001). Subgroup analyses revealed similar patterns, with a stronger association observed in participants with diabetes for ≥10 years relative to those with shorter disease (P for interaction = 0.036).

**Conclusion:**

This study highlights a positive VAI-DKD association in elderly T2DM patients, with higher VAI independently linked to an elevated risk of DKD, particularly in those with a longer history of diabetes. More prospective work is necessary to confirm these findings.

## 1 Introduction

Diabetes is a growing global concern, with projections estimating that 643 million individuals will be affected by 2030 (1). Among these, up to 35% are expected to develop diabetic kidney disease (DKD), a major contributor to chronic kidney disease (CKD) as well as to the incidence of end-stage kidney disease (ESKD), which often require dialysis or transplantation (2). Type 2 diabetes mellitus (T2DM) often coincides with DKD in older patients (3), affecting 32% of those aged 65–75 and 61% of individuals older than 75 (4). Managing DKD in older adults is particularly critical due to its significant health consequences and economic burden. Identifying modifiable risk factors is essential for prevention and improved clinical outcomes.

The rising prevalence of obesity further compounds the risk of diabetes and kidney disease (5–7). Although previous studies have primarily focused on body mass index (BMI) as a measure of obesity, evidence suggests that visceral obesity, as opposed to generalized fat deposition, is more strongly linked with DKD (8–11). While advanced imaging modalities provide accurate assessments of visceral fat, their cost renders them impractical for routine clinical use. The visceral adiposity index (VAI), an indicator of visceral fat level calculated using triglycerides (TG), waist circumference (WC), BMI, and high-density lipoprotein cholesterol (HDL-C) through sex-specific formulas (12), has emerged as a different means of gauging visceral fat accumulation and dysfunction (13). VAI is also linked to metabolic disorders, such as cardiovascular disorders (14), hypertension (15), and diabetes (16). It is also positively associated with declining renal function (17), albuminuria (18–20), and CKD (21). While some studies have reported a link between VAI and DKD (22, 23), there is limited evidence in elderly populations. There is thus a need for further analyses to evaluate the usefulness of VAI in DKD among older adults.

Here, the association between VAI and DKD in elderly T2DM patients was investigated, providing insights that may facilitate early detection and treatment of DKD.

## 2 Materials and methods

### 2.1 Study subjects

In total 13,017 diabetes patients were initially screened at the MMCs of Yuhuan Second People’s Hospital and Taizhou Central Hospital (Taizhou University Hospital) between September 2017 and May 2024. We excluded individuals with (1) T1DM or other forms of diabetes, (2) younger than 65 years, (3) missing values, such as urinary albumin-to-creatinine ratio (UACR), serum creatinine (Scr), WC, TG, HDL-C, and glycated hemoglobin (HbA1c) data. Ultimately, 2,695 patients were incorporated into the final analysis. The Clinical Research Ethics Committees of both hospitals provided protocol approval. As per the Declaration of Helsinki, all subjects gave written informed consent.

### 2.2 Data collection

Comprehensive clinical information was obtained from all participants through institutional electronic medical records by trained interviewers following standardized protocols. Demographic factors, such as age, sex, and educational level, were analyzed. Medical factors encompassed duration of diabetes, presence of diabetes in the family, hypertension, dyslipidemia, coronary heart disease, and stroke, while lifestyle information included smoking and drinking. Physical examination data, including height, weight, WC, and blood pressure, were documented. Laboratory measurements included fasting blood glucose (FBG), fasting serum C-peptide (FCp), HbA1c, total cholesterol (TC), TG, HDL-C, LDL-C, Scr, urea nitrogen (UN), uric acid (UA), and UACR, all performed at the respective hospitals. BMI was weight (kg) divided by height squared (m^2^). Insulin resistance was determined with the homeostasis model assessment of insulin resistance (HOMA-IR) formula: 1.5 + (fasting C peptide [pmol/L] × fasting glucose [mmol/L]) /2800 (24). All estimated glomerular filtration rate (eGFR) calculations were made utilizing the formula from the Chronic Kidney Disease Epidemiology Collaboration (25).

### 2.3 Definition of VAI and DKD

The VAI was computed with equations incorporating WC, BMI, TG, and HDL-C levels that were gender-specific (12).

VAI calculations among males were performed as follows:

$\begin{array}{l} \mathrm{VAI}=\mathrm{WC}(\mathrm{cm})/[39.68+1.88\times \mathrm{BMI}(\mathrm{kg}/m^{2})]\\ \times [\mathrm{TG}(\text{mmol}/L)/1.03]\times [1.31/\mathrm{HDL}(\text{mmol}/L)] \end{array}$

VAI calculations among females were performed as follows:

$\begin{array}{l} \mathrm{VAI}=\mathrm{WC}(\mathrm{cm})/[36.58+1.89\times \mathrm{BMI}(\mathrm{kg}/m^{2})]\\ \times [\mathrm{TG}(\text{mmol}/L)/0.81]\times [1.52/\mathrm{HDL}(\text{mmol}/L)] \end{array}$

DKD was diagnosed by identifying those T2DM patients with a UACR > 30 mg/g and/or an eGFR < 60 mL/min/1.73m^2^ (26).

### 2.4 Statistical analyses

Normally distributed and skewed data are, respectively, reported as means (SD) and medians (IQR), comparing the data with one-way ANOVAs and Kruskal-Wallis H tests, respectively, while categorical data are shown as frequencies (%) and analyzed among VAI tertiles with χ^2^ tests. Univariate and multivariate binary logistic regression was utilized to examine the VAI-DKD link, using three levels of adjustment: Model 1, adjusted for age and sex; Model 2, with additional adjustments for HbA1c, HOMA-IR, diabetes duration, and education; Model 3, with further adjustments for dyslipidemia, hypertension, smoking, drinking, coronary heart disease, and stroke. VAI was analyzed as a continuous variable, and to examine nonlinearity, was also assessed as a categorical variable with tertiles. A P for trend was used to confirm consistency across the models. To explore possible nonlinear dose–response relationships between VAI and DKD, restricted cubic spline (RCS) models were applied. Subgroup and interaction analyses were performed to investigate effect modifications based on the following variables: sex, age (<75 or ≥75 years), diabetes duration (<10 or ≥10 years), HbA1c (<7.5% or ≥7.5%), presence of hypertension, and presence of dyslipidemia. Participants were then stratified into KDIGO risk categories (27) using combined eGFR and UACR values for sensitivity analyses: low risk, moderate risk, high risk and very high risk. R software (version 4.2.2, http://www.Rproject.org, The R Foundation) and Free Statistics software version 2.0 were employed for all analyses, treating a *p* < 0.05 as significant.

## 3 Results

### 3.1 Baseline characteristics of the study population

**Figure 1:**
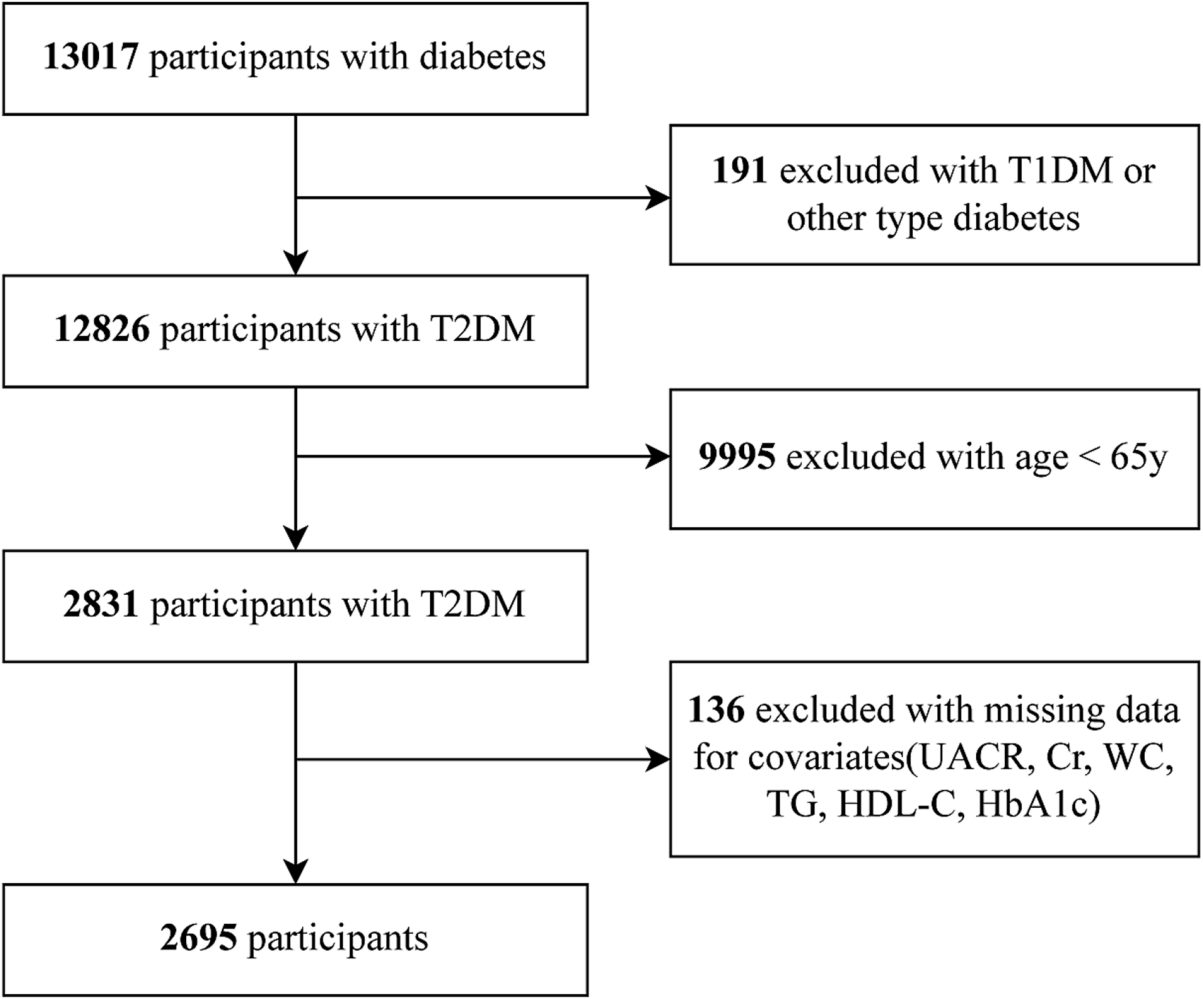
The flow chart of the study.

The flow chart of our study is showed in Figure 1. Table 1 shows participant information, stratified by VAI tertiles. The median age was consistent across all tertiles (*p* = 0.517). However, the proportion of male participants decreased significantly with increasing VAI (60.5% in T1 vs. 33.4% in T3, *p* < 0.001). Smoking and drinking were more common in the lowest VAI tertile (both *p* < 0.001), while the highest VAI tertile was linked to markedly greater BMI, DBP, SBP, FBG, fasting C-peptide (FCp), HOMA-IR, HbA1c, TG, and LDL-C (all *p* < 0.001). Conversely, HDL-C levels decreased as VAI increased (*p* < 0.001). Renal function markers revealed a marked decreases in eGFR and increases in the UACR in the higher tertiles (both *p* < 0.001). Hypertension and dyslipidemia rates also showed progressive increases in relation to VAI (both *p* < 0.001). In contrast, the rates of stroke and coronary heart disease were comparable across the tertiles (*p* = 0.912 and *p* = 0.920, respectively). Notably, DKD levels increased progressively with higher VAI, rising from 48.1% in T1 to 57.6% in T3 (*p* < 0.001).

**Table 1:** Baseline characteristics of the study participants.

Variables	VAI				
	Total (*n* = 2,695)	T1 (*n* = 898)	T2 (*n* = 898)	T3(*n* = 899)	*p* value
Age (y)	70.0 (67.0, 73.0)	69.0 (67.0, 73.0)	70.0 (67.0, 73.0)	69.0 (67.0, 73.0)	0.517
Males, *n* (%)	1,203 (44.6)	543 (60.5)	360 (40.1)	300 (33.4)	< 0.001
Education, *n* (%)					0.392
Below high school	2,593 (96.3)	867 (96.5)	868 (96.7)	858 (95.5)	
High school education and above	101 (3.7)	31 (3.5)	30 (3.3)	40 (4.5)	
Duration of diabetes (y)	9.3 (2.4, 14.3)	9.5 (2.4, 15.2)	8.8 (2.2, 14.0)	9.4 (2.7, 14.1)	0.151
Family history of diabetes, *n* (%)	1,011 (37.5)	341 (38)	335 (37.3)	335 (37.3)	0.941
Smoking, *n* (%)	364 (13.6)	155 (17.4)	105 (11.7)	104 (11.6)	< 0.001
Drinking, *n* (%)	201 (7.5)	104 (11.7)	53 (5.9)	44 (4.9)	< 0.001
SBP (mmHg)	133.0 (122.0, 146.0)	131.0 (120.2, 144.0)	134.0 (122.0, 146.0)	134.0 (124.0, 148.0)	0.001
DBP (mmHg)	71.0 (64.0, 77.0)	70.0 (62.0, 76.0)	71.0 (63.0, 78.0)	72.0 (65.0, 78.0)	< 0.001
BMI (kg/m2)	24.8 (22.7, 27.1)	23.9 (21.9, 26.2)	24.9 (23.0, 27.4)	25.5 (23.7, 27.6)	< 0.001
VAI	2.0 (1.3, 3.3)	1.0 (0.8, 1.3)	2.0 (1.7, 2.4)	4.1 (3.3, 5.5)	< 0.001
FBG (mmol/L)	7.8 (6.2, 9.9)	7.6 (6.1, 9.6)	7.6 (6.1, 9.5)	8.2 (6.6, 10.4)	< 0.001
FCp(ng/mL)	2.1 (1.5, 2.9)	1.6 (1.1, 2.3)	2.2 (1.6, 2.9)	2.5 (1.8, 3.4)	< 0.001
HOMA-IR	3.5 (2.8, 4.5)	3.0 (2.5, 3.8)	3.4 (2.8, 4.3)	4.0 (3.2, 5.1)	< 0.001
HbA1c (%)	7.8 (6.8, 9.5)	7.7 (6.7, 9.5)	7.6 (6.7, 9.3)	8.2 (7.0, 9.7)	< 0.001
UN (mmol/L)	6.0 (4.9, 7.5)	6.0 (5.0, 7.4)	6.0 (4.8, 7.5)	6.0 (4.9, 7.5)	0.558
Scr (mmol/L)	67.0 (55.0, 85.0)	66.0 (55.0, 82.0)	67.0 (54.0, 85.0)	68.0 (56.0, 89.0)	0.023
e-GFR (mL/min per 1.73 m2)	86.7 (68.2, 105.8)	91.8 (74.4, 111.7)	86.0 (67.6, 104.6)	82.0 (62.8, 99.5)	< 0.001
UA (mmol/L)	324.0 (266.0, 399.0)	303.0 (251.0, 365.8)	323.0 (268.0, 398.8)	346.5 (281.0, 431.8)	< 0.001
TG (mmol/L)	1.4 (0.9, 1.9)	0.8 (0.7, 1.0)	1.4 (1.1, 1.6)	2.2 (1.8, 2.9)	< 0.001
TC (mmol/L)	4.8 (4.0, 5.7)	4.6 (3.8, 5.4)	4.8 (4.0, 5.7)	5.0 (4.1, 5.9)	< 0.001
HDL-C (mmol/L)	1.1 (0.9, 1.3)	1.4 (1.2, 1.6)	1.1 (1.0, 1.3)	0.9 (0.8, 1.1)	< 0.001
LDL-C (mmol/L)	2.7 (2.0, 3.4)	2.6 (1.9, 3.2)	2.8 (2.1, 3.6)	2.6 (2.0, 3.3)	< 0.001
UACR(mg/g)	26.7 (11.5, 81.4)	24.8 (10.9, 77.6)	25.4 (10.8, 68.0)	31.9 (12.8, 98.5)	< 0.001
Hypertension, *n* (%)	1862 (69.1)	549 (61.1)	636 (70.8)	677 (75.3)	< 0.001
Dyslipidemia, *n* (%)	744 (27.6)	195 (21.7)	244 (27.2)	305 (33.9)	< 0.001
Coronary heart disease, *n* (%)	314 (11.7)	108 (12)	103 (11.5)	103 (11.5)	0.912
Stroke, *n* (%)	273 (10.1)	92 (10.2)	88 (9.8)	93 (10.3)	0.92
DKD, *n* (%)	1,407 (52.2)	432 (48.1)	457 (50.9)	518 (57.6)	< 0.001

Data are presented as medians (interquartile ranges) or counts(%).

VAI, visceral adiposity index; T, tertile; SBP, systolic blood pressure; DBP, diastolic blood pressure; BMI, body mass index; FBG, fasting blood glucose; FCp, fasting serum C peptide; HOMA-IR, homeostasis model assessment of insulin resistance; HbA1c, glycated hemoglobin; UN, urea nitrogen; Scr, serum creatinine; eGFR, estimated glomerular filtration rate; UA, uric acid; TG, triglycerides; TC, total cholesterol; HDL-C, high-density lipoprotein cholesterol; LDL-C, low-density lipoprotein cholesterol; UACR, urinary albumin-to-creatinine ratio; DKD, diabetic kidney disease.

### 3.2 Association between VAI and DKD in elderly T2DM patients

**Figure 2:**
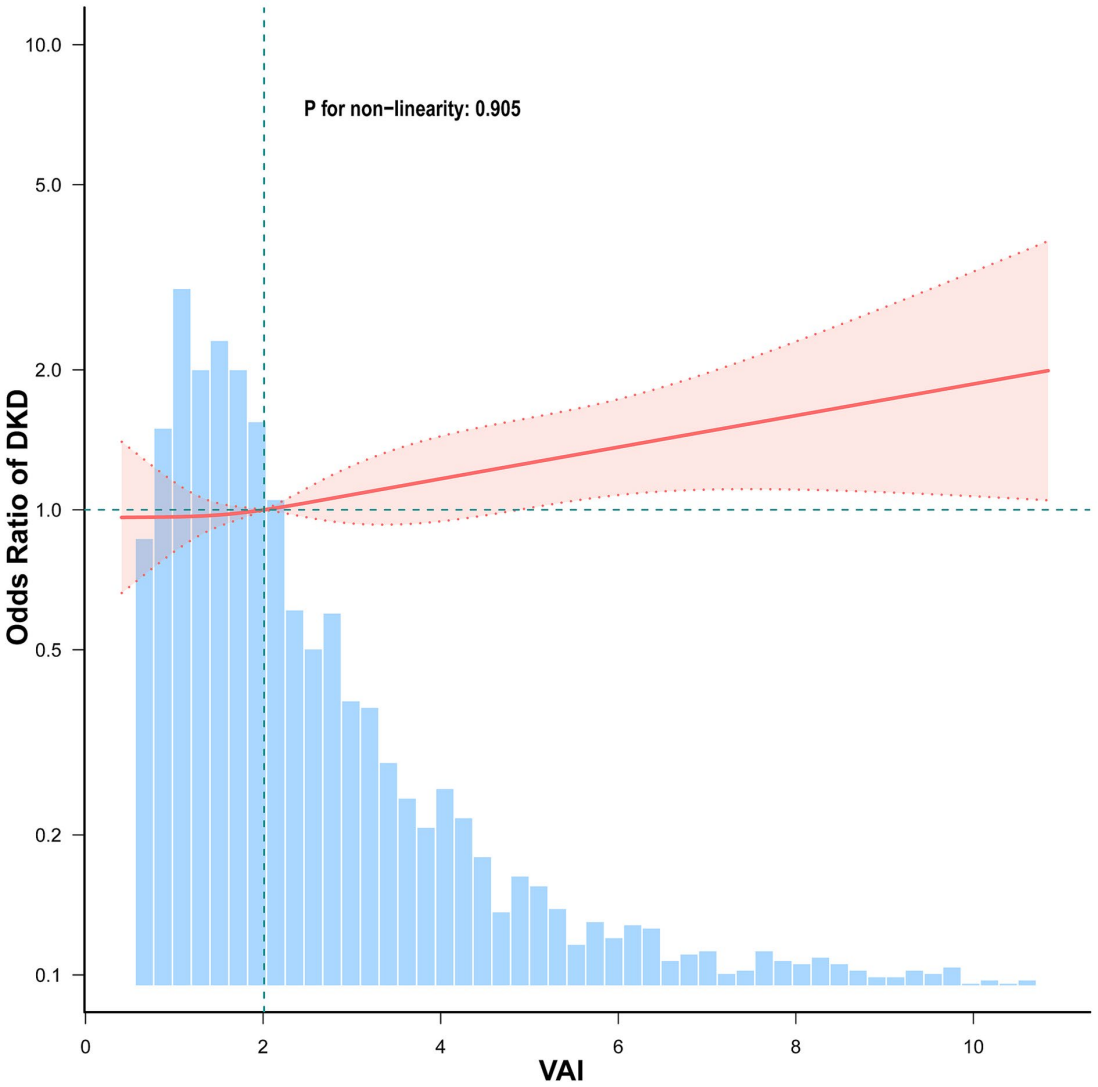
Association between VAI and DKD among elderly T2DM patients. Adjusted for sex, age, HbA1c, HOMA-IR, duration of diabetes, education, SBP, smoking, drinking, hypertension, dyslipidemia, coronary heart disease, and stroke. The red solid line represents the fitted smooth curve for the association between VAI and the OR of DKD, while the shaded pink region denotes the 95% confidence interval (CI). The blue histogram indicates the frequency distribution of VAI in the study population.

Figure 2 illustrates the presence of a positive linear association between VAI and DKD such that ORs rose progressively as VAI levels increased (P for non-linearity = 0.905).

Multivariate logistic regression was utilized to examine the VAI-DKD link in elderly T2DM patients, with VAI used as a continuous and a categorical variable (Table 2). As the former, higher VAI showed a marked association with higher DKD risk in all models. In Model 3, with full adjustment, an increase of one VAI unit led to a 4% greater chance of developing DKD (OR = 1.04, 95%CI: 1.01–1.08, *p* = 0.015). Furthermore, when categorized into tertiles, participants in T3 had markedly greater risks of developing DKD relative to those in T1. The crude model showed that the risk in T3 was 1.47 times higher than T1 (OR = 1.47, 95%CI: 1.22–1.77, *p* < 0.001). Following confounder adjustments, the association remained statistically significant in Model 3, with T3 exhibiting 1.29-fold greater odds of DKD relative to T1 (OR = 1.29, 95%CI: 1.05–1.59, *p* = 0.015). In addition, the trend analysis confirmed VAI and DKD risk to be significantly positively related across all models (P for trend < 0.05).

**Table 2:** Multivariate analysis of association between VAI and DKD.

Variable	Crude		Model 1		Model 2		Model 3	
	OR (95%CI)	*p* value	OR (95%CI)	*p* value	OR (95%CI)	*p* value	OR (95%CI)	*p* value
Continuous	1.07 (1.03–1.1)	<0.001	1.07 (1.04–1.11)	<0.001	1.05 (1.01–1.08)	0.009	1.04 (1.01–1.08)	0.015
Categories								
T1	1(Ref)		1(Ref)		1(Ref)		1(Ref)	
T2	1.12 (0.93–1.35)	0.238	1.13 (0.93–1.36)	0.215	1.08 (0.89–1.32)	0.418	1.06 (0.87–1.29)	0.595
T3	1.47 (1.22–1.77)	<0.001	1.51 (1.25–1.83)	<0.001	1.33 (1.08–1.63)	0.006	1.29 (1.05–1.59)	0.015
P for trend		<0.001		<0.001		0.006		0.015

Model 1: Adjusted for sex, age; Model 2: Adjusted for Model 1 + HbA1c, HOMA-IR, duration of diabetes, education; Model 3: Adjusted for Model 2 + smoking, drinking, hypertension, dyslipidemia, coronary heart disease, stroke.

VAI, visceral adiposity index; DKD, diabetic kidney disease; OR, odds ratio; CI, confidence interval; T, tertile; HOMA-IR, homeostasis model assessment of insulin resistance; HbA1c, glycated hemoglobin.

### 3.3 Subgroup analysis

**Figure 3:**
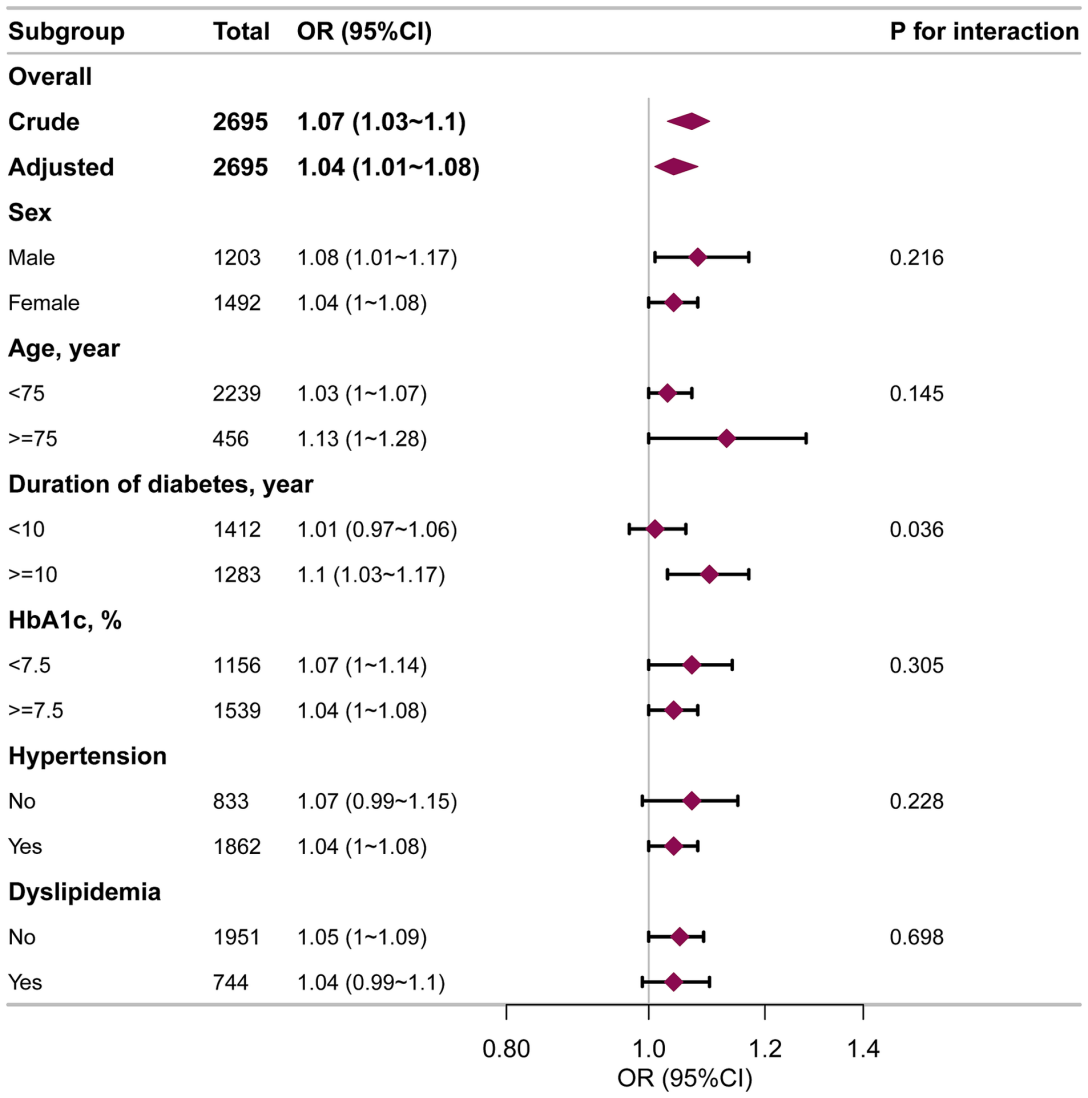
Subgroup analysis of the VAI and DKD among elderly T2DM patients. Each stratification factor was adjusted for age, sex, HbA1c, HOMA-IR, duration of diabetes, education, smoking, drinking, hypertension, dyslipidemia, coronary heart disease, and stroke.

Subgroup analyses were employed to investigate if the VAI-DKD association varied by specific characteristics (Figure 3). Across all subgroups, higher VAI levels were independently linked to an increased DKD risk in both crude and fully adjusted models. No significant interactions were observed for sex, age (<75 years vs. ≥75 years), HbA1c (<7.5% vs. ≥7.5%), hypertension, or dyslipidemia. However, diabetes duration significantly modified the VAI-DKD relationship (P for interaction = 0.036). In patients who had suffered from diabetes for ≥10 years, elevated VAI was strongly linked with greater DKD likelihood (OR = 1.10, 95%CI: 1.03–1.17). In contrast, this link was not seen in individuals with a < 10-year diabetes duration (OR = 1.01, 95%CI: 0.97–1.06).

**Figure 4:**
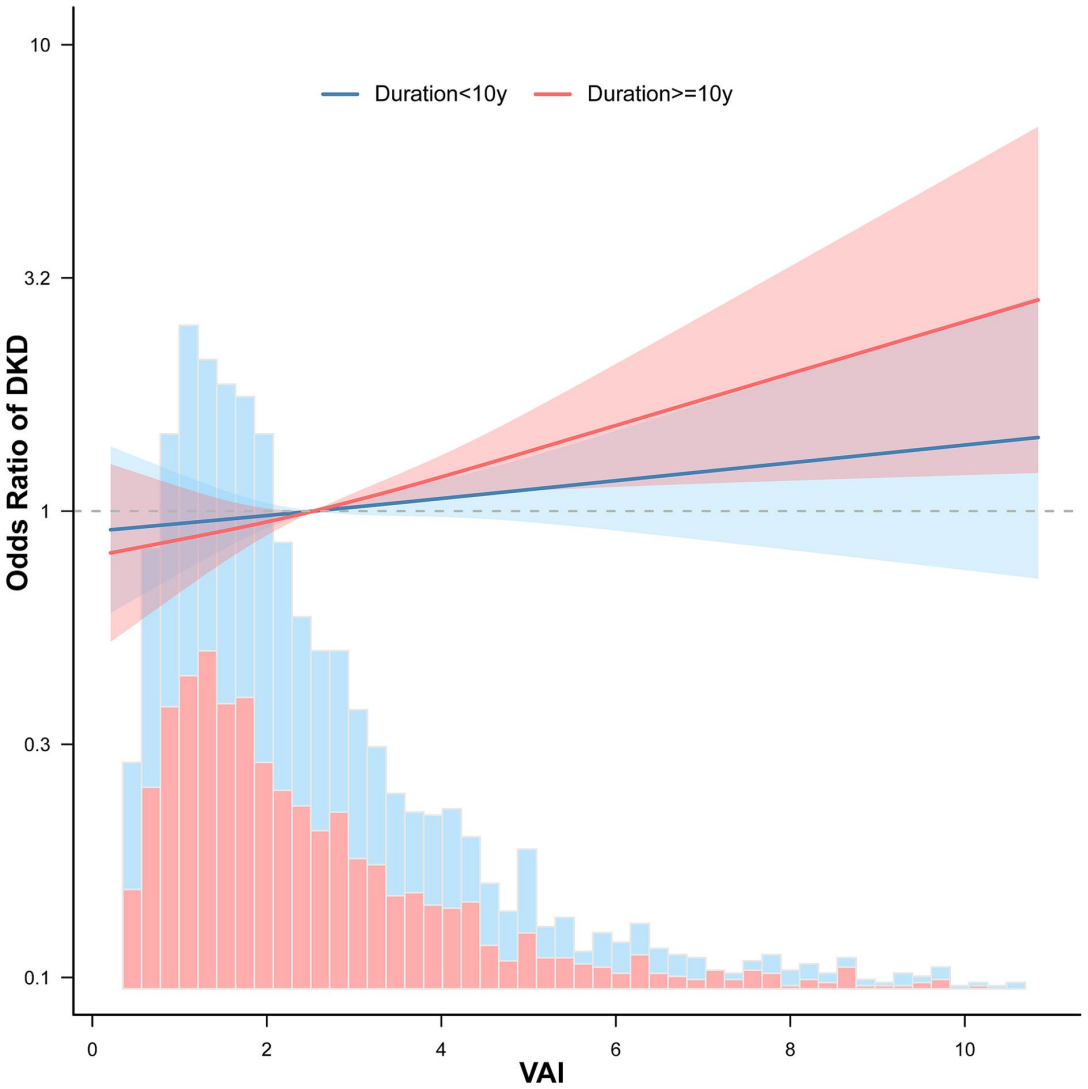
Association between VAI and DKD among elderly T2DM patients stratified by diabetes duration. ORs for DKD with 95% CIs are shown for two subgroups based on diabetes duration: <10 years (blue) and ≥10 years (red). The solid lines represent smoothed ORs, while the shaded areas indicate 95% CIs.

Further analysis of diabetes duration revealed distinct trends in the link between VAI and DKD (Figure 4). For those who had suffered from diabetes for <10 years, there was a slight rise in the DKD risk with increased VAI. However, in cases who had had diabetes for ≥10 years, the chance of DKD increased markedly as VAI levels rose.

### 3.4 Sensitivity analysis

To back up our conclusions, we conducted sensitivity analyses. The DKD patients were futher divided into four stages according to the KDIGO risk categories as follows: low risk, moderate risk, high risk and very high risk. With increasing KDIGO risk severity, VAI levels showed a gradual upward trend (*p* < 0.001, Supplementary Figure S1). The proportion of patients in the very high risk category increases with higher VAI tertiles (Supplementary Figure S2).

Table 3 shows the relationship between VAI and DKD stages. In the crude model, compared with the low-risk DKD stage, each 1-unit increase in VAI was associated with a 4% increase in the risk of intermediate-risk DKD stage (OR = 1.04, 95% CI: 1–1.08, *p* = 0.05), a 9% increase in high-risk DKD stage (OR = 1.09, 95% CI: 1.04–1.14, *p* < 0.001), and a 13% increase in very high-risk DKD stage (OR = 1.13, 95% CI: 1.08–1.18, *p* < 0.001). After adjusting for potentially important confounders, as VAI increased, the risk of DKD was not significant in the moderate risk group (*p* > 0.05), meanwhile, it was significantly increased in the high risk and very high risk group (*p* < 0.01).

**Table 3:** Effect-size estimates of VAI with KDIGO Risk Categories of DKD.

DKD risk	Low risk	Moderate risk		High risk		Very high risk	
		OR (95%CI)	*p* value	OR (95%CI)	*p* value	OR (95%CI)	*p* value
Crude	Reference	1.04 (1 ~ 1.08)	0.05	1.09 (1.04 ~ 1.14)	<0.001	1.13 (1.08 ~ 1.18)	<0.001
Model 1	Reference	1.04 (1 ~ 1.08)	0.059	1.1 (1.05 ~ 1.15)	<0.001	1.14 (1.09 ~ 1.2)	<0.001
Model 2	Reference	1.02 (0.98 ~ 1.06)	0.398	1.08 (1.03 ~ 1.13)	0.002	1.12 (1.07 ~ 1.18)	<0.001
Model 3	Reference	1.02 (0.98 ~ 1.06)	0.325	1.08 (1.03 ~ 1.13)	0.002	1.13 (1.08 ~ 1.18)	<0.001

Model 1: Adjusted for sex, age; Model 2: Adjusted for Model 1 + HbA1c, HOMA-IR, duration of diabetes, education; Model 3: Adjusted for Model 2 + smoking, drinking, hypertension, dyslipidemia, coronary heart disease, stroke.

VAI, visceral adiposity index; DKD, diabetic kidney disease; OR, odds ratio; CI, confidence interval; HOMA-IR, homeostasis model assessment of insulin resistance; HbA1c, glycated hemoglobin.

## 4 Discussion

In our study, the VAI-DKD relationship was explored among elderly patients with T2DM. We detected a significant positive correlation linking VAI to DKD, with this association being more pronounced in individuals with a longer diabetes duration.

Previous research has identified VAI as a marker linked to various metabolic disorders. For example, a prospective study demonstrated VAI to be independently and dose-dependently associated with NAFLD risk (28). Data from the NHANES surveys revealed positive nonlinear correlations between VAI and both prediabetic and diabetic risk among American adults (29). Motamed et al. (30) highlighted VAI’s diagnostic value for metabolic syndrome. Moreover, several studies have established a connection between elevated VAI and kidney disease. Xiao et al. (21) observed a positive link between VAI and CKD among individuals without diabetes, as well as an inverse relationship with eGFR. Similarly, an analysis of NHANES 2011–2018 data indicated a subtantial correlation between VAI and CKD in Americans 60 + years old (31). Other studies have shown elevated VAI levels to be linked to reduced renal function (17) and increased proteinuria (18–20).

Here, we confirmed a positive VAI-DKD relationship in elderly T2DM patients, consistent with previous findings. For instance, Li et al. (32) analyzed data from 2,508 diabetes cases from the NHANES surveys, finding that DKD patients had raised VAI levels relative to non-DKD patients. After adjusting for confounders, VAI and DKD were positively associated (OR = 1.050, 95% CI 1.049–1.050), with cases in the top VAI tertile showing a 35.9% greater DKD risk (OR = 1.359, 95% CI 1.355–1.362). Our study similarly found an OR of 1.29 (95% CI 1.05–1.59) for the highest VAI tertile. Wu et al.’s (23) reported that DKD risk was amplified with rising VAI levels (HR = 1.127, 95% CI 1.050–1.210), and Sun et al. (33) reported a substantial link between VAI and DKD risk following confounder adjustment (HR = 1.132, 95% CI 1.001–1.281). Additionally, Zhao et al. (34) found VAI and UACR to be positively correlated among T2DM patients, with VAI levels rising alongside DKD severity. Zhou et al. (35) further reported that higher VAI levels were related to greater risk of nephropathy in T2DM, independent of its components such as BMI, HDL-C, and triglycerides. Numerous other studies also support the significant relationship between VAI and DKD (22, 36–38).

Despite these similarities, our findings diverge from some prior research. For example, Wan et al. (22) reported that a one-SD rise in VAI was linked to DKD prevalence in women (OR = 1.51; 95% CI 1.29–1.76, *p* < 0.05), while the same was not evident in men. Conversely, Li et al.’s (32) found that the significant VAI-DKD relationship was only evident in males. In our study, however, the consistent VAI-DKD relationship was evident across genders. These discrepancies may stem from variations in terms of region, ethnicity, sample size, and design of the study. Another notable finding of our study is the stronger positive VAI-DKD association among patients with a longer duration of diabetes. While this observation aligns with previous evidence suggesting that disease duration is an important modifier of DKD risk, further research is crucial to confirm this hypothesis and shine light on the underlying mechanisms.

These findings provide valuable insight into the interplay between VAI and DKD in elderly T2DM patients. Higher VAI was found to be independently linked to greater DKD risk, highlighting visceral fat as an important risk for renal complications in this population. From a clinical perspective, VAI holds promise as a useful biomarker for identifying patients at risk of DKD, particularly in resource-limited settings where advanced imaging techniques or expensive biomarkers may be unavailable. Compared to prior studies, our research utilized a large dataset of 2,695 participants from two hospitals, enhancing both the generalizability and statistical power of the results. Additionally, adjustments were made for numerous covariates, including laboratory markers, lifestyle factors, and comorbidities, and stratified and interaction analyses were performed to provide subgroup-specific insights. These robust methodological approaches strengthen the validity of our results. Given the increasing prevalence of diabetes and obesity worldwide, incorporating VAI into routine clinical assessments could facilitate early and personalized interventions to improve DKD outcomes. Further investigations should focus on determining the biological mechanisms linking VAI and DKD, conducting longitudinal studies to establish causality, and evaluating the effectiveness of interventions targeting visceral fat in reducing DKD risk.

The association between the VAI and DKD in elderly patients with T2DM can be attributed to multiple interrelated factors. Visceral fat secretes pro-inflammatory cytokines and adipokines (39), which can induce systemic inflammatory responses, insulin resistance, and glucose metabolism disorders, thereby accelerating the progression of diabetes and its complications. Additionally, increased visceral fat may directly impair renal function by causing inflammation and fibrosis in renal microvasculature, affecting renal hemodynamics, and increasing urinary protein excretion (40). The close relationship between visceral fat and metabolic syndrome may further exacerbate renal damage through factors such as hyperglycemia, hypertension, and dyslipidemia. Moreover, visceral fat accumulation is associated with elevated oxidative stress levels (41), which can cause cellular damage and inflammatory responses, ultimately contributing to renal injury. These mechanisms collectively underlie the association between VAI and DKD.

This study has several limitations. For one, causal inferences are not possible given its cross-sectional design, underscoring the need for prospective cohort studies to explore causal associations between VAI and DKD. Second, the study included hospital-based patients, potentially reducing the generalizability of the results. Finally, despite adjustments for numerous potential confounders, factors such as physical activity, dietary habits, and medication use were not accounted for, leaving room for residual confounding.

## 5 Conclusion

In summry, a significant positive between was found between VAI and DKD in elderly T2DM cases. Higher VAI is independently linked with an elevated chance of DKD development, especially in the case of a long history of diabetes. These results suggest the potential of using VAI in the clinic to identify high-risk patients and emphasize that targeted interventions are necessary to prevent DKD. Further investigations are essential to verify these results and clarify the mechanistic basis.

## Data Availability

The raw data supporting the conclusions of this article will be made available by the authors, without undue reservation.
