# Supplementary figures and images for: The association of visceral adiposity index and diabetic kidney disease in elderly patients with type 2 diabetes mellitus: a cross-sectional study
Source: Front Nutr. 2025 Jun 20;12:1556886. doi: 10.3389/fnut.2025.1556886 (PMC12231457; doi:10.3389/fnut.2025.1556886)

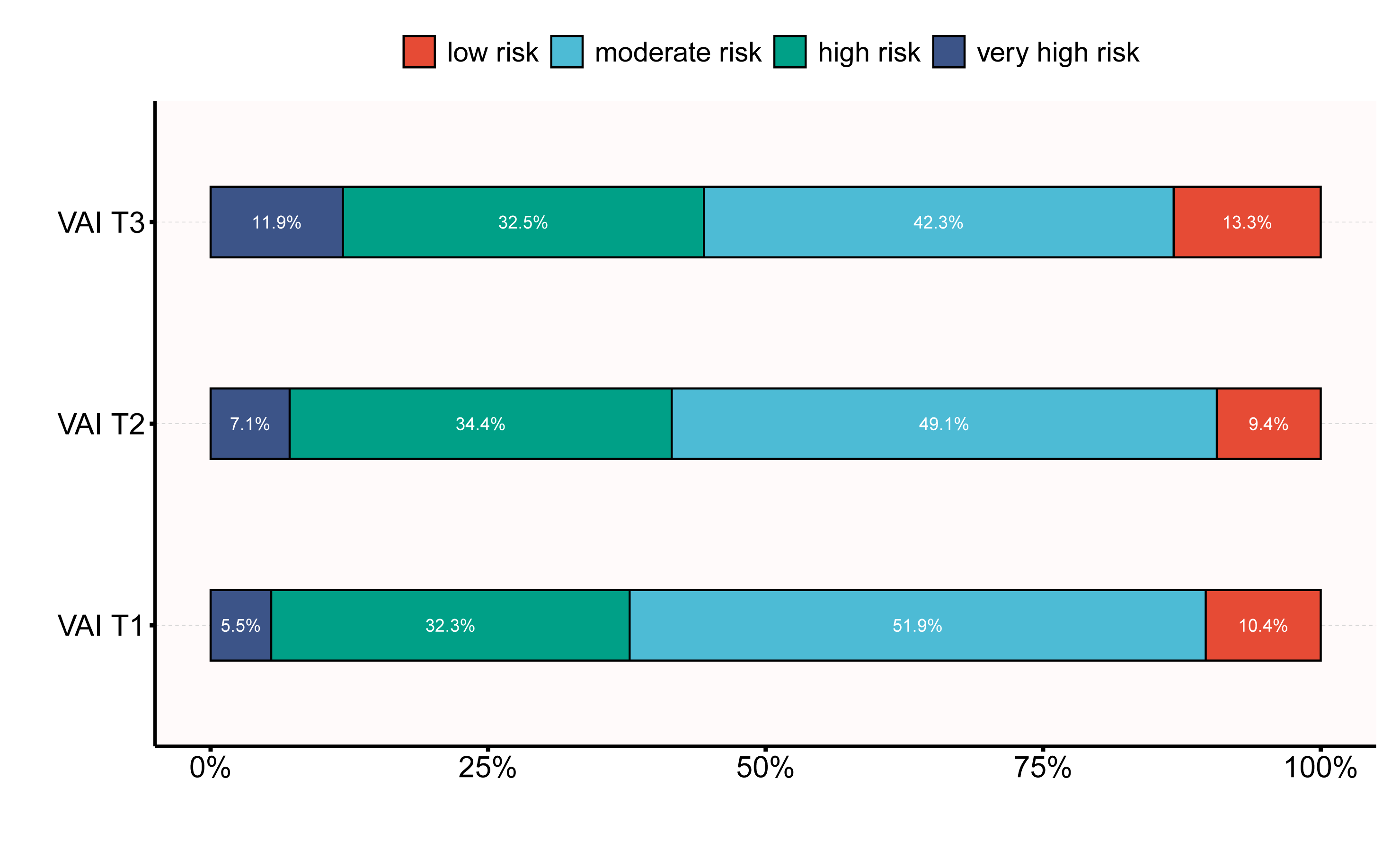

Supplement: Supplementary file 1 [file Image_1.TIF]

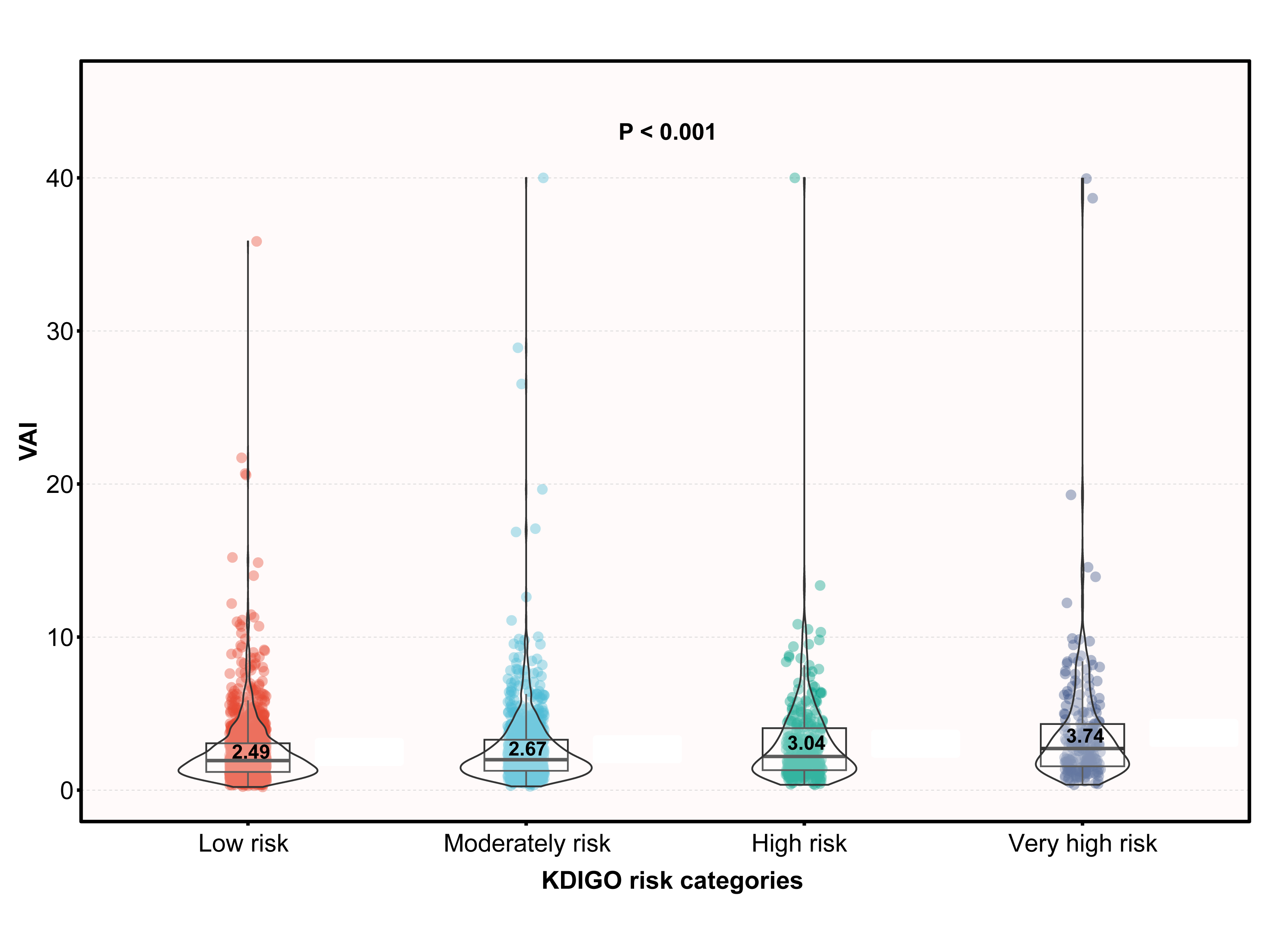

Supplement: Supplementary file 2 [file Image_2.TIF]
